# Prevalence and Molecular Characterization of Plasmid-mediated Extended-Spectrum β-Lactamase Genes (bala_TEM_, bla_CTX _and blA_SHV_) Among Urinary *Escherichia coli* Clinical Isolates in Mashhad, Iran

**Published:** 2012

**Authors:** Mahboobeh Nakhaei Moghaddam, Mohammad Mahdi Forghanifard, Sheila Moshrefi

**Affiliations:** 1 *Department of Biology, Mashhad Branch, Islamic Azad University, Mashhad, Iran*; 2 *Department of Biology, Damghan Branch, Islamic Azad University, Damghan, Iran*

**Keywords:** Antibiotic resistance, Escherichia coli, Extended-spectrum beta-lactamase, Urinary tract infection

## Abstract

**Objective(s):**

Extended-spectrum beta-lactamase (ESBL) producing bacteria have an important role in nosocomial infections. Due to the limited availability of information about the molecular epidemiology of ESBL producing bacteria in Mashhad, we decided to investigate about TEM, CTX and SHV ESBLs among urinary *Escherichia coli* isolates in Mashhad, a city in northeast Iran.

**Materials and Methods:**

One hundred and eleven clinical isolates of *E. coli* were diagnosed from hospitalized patients in 2009. After performing antibiogram and phenotypic confirmation test, polymerase chain reaction (PCR) was performed by bla_TEM_, bla_SHV_ and bla_CTX_ primers and restriction digestion was carried out using PstI and TaqI (Fermentas-Lithuania) for confirmation.

**Results:**

ESBL producers of E*. coli *isolates were 33.3%. Among 37 ESBL-producing isolates, 35 (94.6%), 21 (56.8%) and 5 (13.5%) were shown to have bla_CTX_, bla_TEM_ and bla_SHV_, genes respectively. Co-resistance to non-beta lactam antibiotics was observed more with ESBL producers (*P* < 0.05).

**Conclusion:**

The results showed that the studied ESBL genes are found with high prevalence and among them bla_CTX_ is more widespread in urine *E. coli* isolates in Mashhad.

## Introduction

Production of beta-lactamases is the most important mechanism of resistance to penicillin, cephalosporin, and monobactam (1). Typically, extended-spectrum beta-lactamases (ESBLs) are derived from genes originally encoded for TEM-1, TEM-2, or SHV-1 by point mutations (2, 3) that alter the amino acid configuration around the active site of these beta-lactamases. This extends the spectrum of beta-lactam antibiotics susceptible to hydrolysis by these enzymes (2).

ESBLs are usually described as enzymes that are mediated by genes located on plasmids. Some ESBL-encoding genes are located within transposons or integrons, which facilitate transfer between bacteria. ESBL-producing microorganisms have an important role in nosocomial infections (3, 4). CTX-M beta-lactamases have higher levels of hydrolytic activity against cefotaxime compared to ceftazidime, but are inhibited by clavulanate and tazobactam (4).

TEM, SHV and CTX-M-type of ESBLs are most often found in a wide range of *Enterobacteriaceac* with increasing frequency (7-10). However, the majority of ESBL-producing strains are *Escherichia coli* and *Klebsiella pneumoniae* (11)*.* *E. coli* is an inhabitant of the colon of humans and other warm-blooded animals. Some strains cause gastroenteritis or urinary tract infections (5). *E. coli* is the most common cause of urinary infection (6).

There is limited information about the molecular epidemiology of ESBL in *Enterobacteriaceae* in Mashhad. In the present study we investigated urinary *E. coli* strains isolated from hospitalized patients in two local hospitals. The objectives included the determination of the prevalence of ESBL producers in urinary isolates of *E. coli*, phenotypically and genotypically, and to compare ESBL producers and non-producers susceptibility to non-beta lactam antibiotics.

## Materials and Methods

***Ethical Approval***

This research was conducted in accordance with Ethical Principles on Clinical Specimens and all patients filled out an informed consent.

***Bacterial isolates***

One hundred eleven non-duplicate clinical isolates of *E. coli* from urine samples of hospitalized patients were collected during the 8-month period from February to October of 2009 at the Hefdah- Shahrivar and Ghaem Hospitals in Mashhad.

At first, bacteria were identified using biochemical tests including oxidase, ONPG, indole, H_2_S, gas production from glucose, MRVP, citrate, urease, and malonate. Confirmatory identification was performed by microgen kit (Microgen Bioproducts ID-GNA-UK) beside positive and negative reference strains.

***Antimicrobial susceptibility testing and ESBL detection***

The susceptibility testing of the *E. coli* isolates to antibiotics were examined by agar diffusion method using standard paper disks according to Clinical and Laboratory Standards Institute (CLSI) guidelines (12). Antibiotic susceptibility test was carried out on Mueller-Hinton agar (Merck, Germany) to the fallowing agents: nalidixic acid (NA: 30 µg), imipenem (I: 10 µg), trimethoprim/ sulfamethoxazole (SXT: 25 µg), ciprofloxacin (Cip: 5 µg), gentamicin (G: 10 µg), amikacin (AK: 30 µg), polymyxin (Pb: 300 Iu), nitrofurantoin (F: 300 µg), ceftazidime (CAZ: 30 µg), cefotaxime (Ce: 30 µg) from Liofilichem, Italy and amoxicillin/ clavulanic acid (Augmentin: 10/20 µg) from MAST Diagnostics, UK.

The detection of ESBL-mediated resistance was performed by the double-disk approximation or double disk synergy (DDS) test (13).

ESBL production is inferred when the zone of inhibition around the ceftazidime disk is expanded towards the disk containing clavunate. Phenotypic confirmation of ESBL presence was performed using ceftazidime/clavunate combination disk with ceftazidime according to the British Society for Antimicrobial Chemotherapy (14, 15). An increase of ≥ 5 mm in the zone of the disk containing clavunate compared with the zone diameter of ceftazidime alone indicates the presence of ESBL.

***Plasmid extraction***

To evaluate gene producing ESBL, species plasmid extraction was necessary. Pure colonies were cultured in fresh Lauria Bertani borth (Merck, Germany) containing 100 µg/ml ampicillin. Tubes were incubated at 37º C shaking 185 rmp for 16 hr. Plasmids were extracted using Perfect Prep-Spin Mini Kit (5 Prime-USA) according to procedure guidelines.

To assess correct process of plasmid extraction, the extracted material was run on 2% gel agarose beside DNA size marker (Gene Ruler 100 bp DNA Ladder) and then the gel was stained with ethidium bromide.

***Polymerase chain reaction (PCR) amplification***

Ten µl of plasmid extracts were used as template DNA. PCR was performed in a 30 µl mixture of 3 µl 10x buffer in a thermal cycler (Esco, Singapore). 1 µl of 10 mM, MgCl_2_, 0.25 µl of 5 u/µl Taq DNA polymerase (Fermentas-Lithuania), 0.5 µl of 10 mM of each deoxynucleotide triphosphate, and 1µl of 10 µM of each primer. The PCR mixture was subjected to a 5 min hot start at 94 ºC, followed by 35 cycles at 30 sec at 94 ºC denaturation, 30 sec at 50 ºC (52 ºC bla_TEM_ and 56 ºC for bla_SHV_) for annealing, 30 sec (60 s for bla_SHV_) at 72 ºC for extension, and a final elongation step of 5 min at 72 ºC. The specific primer sets which were used for amplification are shown in Table 1. In this study, bla_CTX_, bla_TEM _and bla_SHV_ producing *E. coli* strains were obtained from *Pasteur Institute* of *Iran* and used as positive controls in PCR assays.

PCR products were electrophoresed in 2% agarose gel and stained with ethidium bromide. A 100 bp DNA ladder (Fermentas, Lithuania) was used as molecular weight marker.

***Confirmation of the amplified products***

The restriction enzymes were selected using CLC Main workbench 5 software. PCR products were extracted with Agarose Gel Extract Mini Kit-50 Prep (5 Prime-USA) according to procedure guidelines. Following PCR, the bla_SHV_ and bla_TEM_ PCR products were digested with PstI (Fermentas-Lithuania) for 3 hr at 37 ºC and bla_CTX_ PCR products were digested with Taq1 (Fermentas-Lithuania) for 3 hr at 65 ºC. For restriction enzyme digestion 6 µl of the each PCR products were mixed with 2.5 µl buffer, 14.5 µl distilled water and 2 µl Taq1 or PstI restriction enzymes.

***Statistical analysis***

Statistical analysis was carried out using Statistica software. Chi-square test used for determination of significance of association. The *P* ≤ 0.05 was considered significant.

## Results

All of 111 *E. coli* isolates were sensitive to imipenem. Isolates exhibited the lowest sensitivity (41.4%) to co-trimoxazole. The antimicrobial susceptibility results of ESBL producers and non-producers are shown in Table 2. There was significant difference between ESBL producing and non-producing isolates for resistance to ceftazidim, cefotaxime, co-trimoxazole, nalidixic acid and ciprofloxacin (*P*< 0.05). More isolates of ESBL producers were found to be resistant to these antibiotics. Nitrofuration resistance was the same between the two groups. More isolates of ESBL producers were resistant to polymyxin and amikacin, however, there was no significant association. Co-resistance to non-beta-lactam antibiotics was observed more (*P*< 0.05) with ESBL producers.

**Table 1:** Primers used for detection of bla genes

Primer	5'-Sequence- 3'	Molecular weight (bp)	Reference No
TEM-F	ACATGGGGGATCATGTAACT	421bp	16
TEM-R	GACAGTTACAATGCTTACT		
SHV-F	ATGCGTTATATTCGCCTGTG	859bp	16
SHV-R	AGCGTTGCCAGTGCTCGATG		
CTX-MU1	ATGTGCAGYACCAGTAARGT	593bp	17
CTX-MU2	TGGGTRAARTARGTSACCAGT		

**Figure 1:**
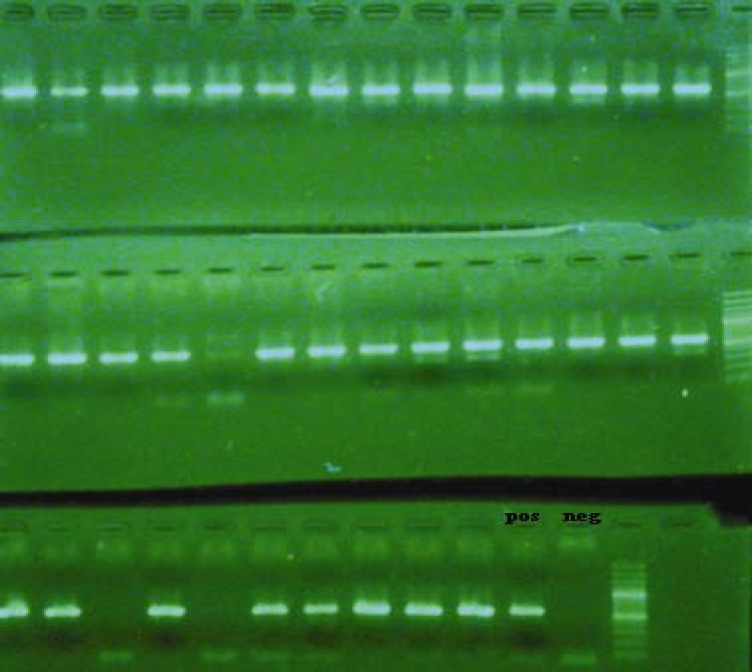
PCR products of bla_CTX_ producing isolates on gel agarose (right lane is marker, 100 bp, pos: positive control, neg: negative control)

ESBL production was observed in 33.3% (37/111) of *E. coli* isolates by approximation and CLSI confirmatory tests. The bla_CTX_ was the most frequent gene (35/37, 94.6%) found in ESBL phenotypic positive isolates using PCR method (Figure 1). Of the 37 ESBL-producing isolates, 21 (56.8%) and 5 (13.5%) were bla_TEM_ and bla_SHV_, respectively. Twenty isolates of ESBL producers co-harbored two of three bla genes and two co-harbored all of three studied bla genes.

**Figure 2:**
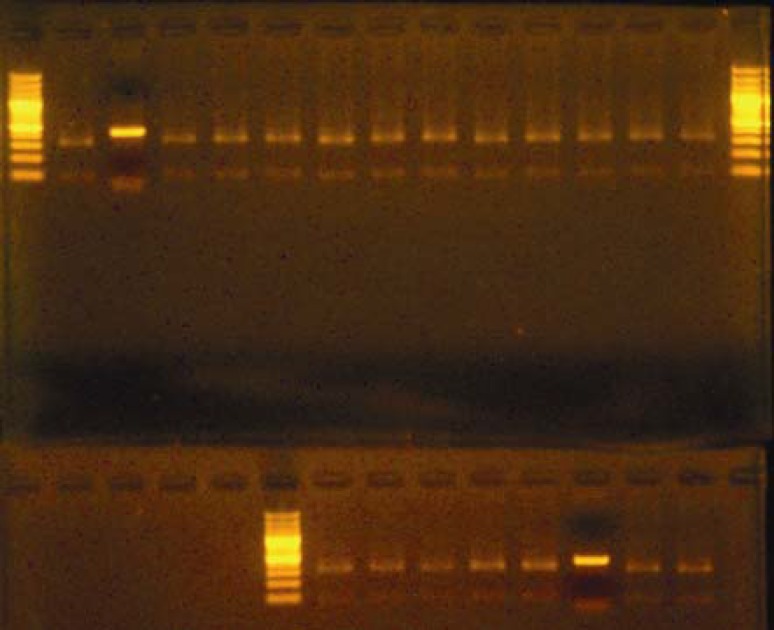
Digestion pattern of the bla_TEM_ amplified products on gel agarose (right and left lanes show marker)

**Figure 3:**
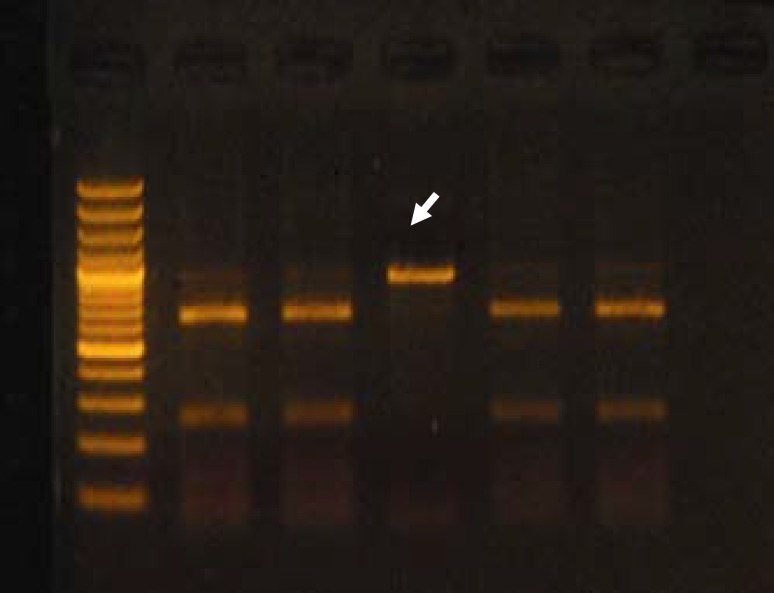
Digestion pattern of the bla_SHV_ amplified products on gel agarose (the arrowhead shows the restriction enzyme pattern of isolate No. 38. left lane shows marker)

The restriction digestion analysis showed that all of the bla_TEM_ amplified products had the same patterns (Figure 2). Therefore, ESBL producers detected by PCR method were confirmed for all isolates carrying bla_TEM_ gene in this study. According to the sequence presented in the NCBI (National Center for Biotechnology Information) website, after enzymatic digestion of bla_TEM_ amplified products by PstI, two fragments of 92 and 330 bp sizes are produced. Also, after digestion of bla_SHV_ amplified products by PstI, two fragments of 615 and 245 bp sizes are generated (Figure 3), while the length of fragments produced after TaqI digestion of bla_CTX_ amplified products are 270 and 323 bp.

Restriction enzyme patterns of isolate No 38 for bla_SHV_ and of isolate No 2 for bla_CTX_ genes were different from the pattern of other isolates. This difference may be due to a mutation in excision site of these enzymes that need to be examined in more details.

## Discussion

ESBL-producing organisms are now increasing among clinical isolates worldwide. Our finding showed that the prevalence of ESBL producing isolates of *E. coli* was 33.3% in studied patients in Mashhad. The prevalence was different between two hospitals (50% in Qhaem Hospital vs. 26.3% in Hefdah- Shahrivar Hospital). The prevalence of ESBL producers varies among clinical isolates from different geographic areas. The presence of ESBL was confirmed in 39% of the *Enterobacteriaceae* isolates resistant to expanded-spectrum cephalosporins in North and center of Portugal (18) over a 2 year period (2002-4) and 37 out of 133 *E. coli *isolates were ESBL producing. About 38-39% of *E. coli *isolates from Emirate (19) in 2005-6 were identified as having ESBL. Much higher prevalence of ESBL has been reported from Latin America: 30-60%, Turkey: 58%, and India: 56% (2, 20). However, low rates (5-8%) of ESBL-producing *E. coli* have been reported in Korea, Japan, Malaysia and Singapore (2, 21-23). Lower than 1% of *E. coli* isolates were reported to be ESBL positive in Netherlands (24).

**Table 2:** Susceptibility results for ESBL-producing and non-ESBL producing *E.coli* strains isolated in this study

	ESBL	-producing	Isolates (%)	ESBL-non	-producing	Isolates (%)
Antibiotic	S	I	R	S	I	R
Cip	19 (51.4)	2 (5.4)	16 (43.2)	67 (90.5)	0 (0)	7 (9.5)
NA	11 (29.7)	2(5.4)	24 (64.9)	59 (79.7)	1(1.4)	14 (18.9)
I	37 (100)	0(0)	0 (0)	74 (100)	0(0)	0 (0)
Ce	5 (13.5)	5 (13.5)	27 (73)	61 (82.4)	12 (16.2)	1 (1.4)
CAZ	10 (27)	12 (32.4)	15(40.5)	68(91.9)	1 (1.4)	5 (6.8)
F	34 (91.9)	2 (5.4)	2 (2.7)	72 (97.3)	0 (0)	2 (2.7)
Pb	33 (89.2)	1 (2.7)	2 (5.4)	67 (90.5)	6 (8.1)	1 (1.4)
Ak	34 (91.9)	1 (2.7)	3 (8.1)	70 (94.6)	3 (4.1)	1 (1.4)
SXT	7 (18.9)	1 (2.7)	29 (78.4)	39 (52.7)	3 (4.1)	31 (41.9)
G	20 (54.1)	7 (18.9)	10 (27)	68 (91.9)	1 (1.4)	5 (6.8)

Cip: Ciprofloxacin, NA: Nalidixic acid, I: Imipenem, Ce: Cefotaxime, CAZ: Ceftazidime, F: Nitrofurantion, Pb: Polymyxin, AK: Amikacin, SXT: Trimethoprim/sulfamethoxazole, G: Gentamicin.

According to a study in the United States of America, ESBL-producing *E. coli* isolates, were associated with resistance to co-trimoxazole, nalidixic acid, gentamicin and ciprofloxacine (25). Our study also showed that a higher percentage of ESBL positive isolates were resistant to ciprofloxacine, nalidixic acid and co-trimoxazole (43.2% versus 6.9%). Co-resistance to gentamicin and ciprofloxacin has been reported in Tanzania (26), to gentamicin, co-trimoxazole and quinolones in India (21, 27) and to quinolones and aminoglycosides in Portugal (28).

Isolation and detection of ESBL-producing strains are essential for the selection of most effective antibiotic for treatment. In this study, all of the ESBL producing isolates were identified as imipenem susceptible.

Our finding showed that CTX-M-type β-lactamases are widespread in Mashhad. The prevalence of CTX-M in France (29) and Portugal (28) were 68% (2007) and 66% (2007) respectively among ESBL positive of *E. coli* isolates. However, we found higher prevalence of CTX-M in Mashhad than these developed countries.

Among *E. coli* isolates in a Swedish Hospital, the frequency of CTX-type, TEM-type and SHV-type enzymes were 92%, 63% and 6%, respectively; a fact which is in accordance with the results of our survey: 94.6%, 56.8% and 13.5%, respectively (30).

Considering available research (3, 4), most of the ESBL production is carried via plasmids and these genes could easily transfer among hospitalized patients (4). This is a major factor for increasing spread of ESBL producers. Therefore, proper management for prescription of antibiotics and also identification of ESBL-producing bacteria in communities are important for prevention. 

One isolate of each bla_CTX_ and bla_TEM_ genes did not have the same digestion pattern as others. Further investigation is required to identify the probable mutation in the area of enzyme excision site.

## Conclusion

Our findings illustrated a higher prevalence of the ESBL carrying *E. coli* in our community, compared to the developed countries as well as the widespread presence the bla_CTX_ in the uropathogenic *E. coli* isolates.
